# Silica ecosystem for synergistic biotransformation

**DOI:** 10.1038/srep27404

**Published:** 2016-06-06

**Authors:** Baris R. Mutlu, Jonathan K. Sakkos, Sujin Yeom, Lawrence P. Wackett, Alptekin Aksan

**Affiliations:** 1 Department of Mechanical Engineering, University of Minnesota, Minneapolis, MN 55455, USA; 2 Department of Biochemistry, Molecular Biology and Biophysics, University of Minnesota, Minneapolis, MN 55455, USA; 3 BioTechnology Institute, University of Minnesota, St Paul, MN 55108, USA

## Abstract

Synergistical bacterial species can perform more varied and complex transformations of chemical substances than either species alone, but this is rarely used commercially because of technical difficulties in maintaining mixed cultures. Typical problems with mixed cultures on scale are unrestrained growth of one bacterium, which leads to suboptimal population ratios, and lack of control over bacterial spatial distribution, which leads to inefficient substrate transport. To address these issues, we designed and produced a synthetic ecosystem by co-encapsulation in a silica gel matrix, which enabled precise control of the microbial populations and their microenvironment. As a case study, two greatly different microorganisms: *Pseudomonas* sp. NCIB 9816 and *Synechococcus elongatus* PCC 7942 were encapsulated. NCIB 9816 can aerobically biotransform over 100 aromatic hydrocarbons, a feat useful for synthesis of higher value commodity chemicals or environmental remediation. In our system, NCIB 9816 was used for biotransformation of naphthalene (a model substrate) into CO_2_ and the cyanobacterium PCC 7942 was used to provide the necessary oxygen for the biotransformation reactions via photosynthesis. A mathematical model was constructed to determine the critical cell density parameter to maximize oxygen production, and was then used to maximize the biotransformation rate of the system.

Pure cultures of naturally-occurring or recombinant microorganisms are used for biotransformation of chemicals at large scale^1^,^2^,^3^, most prominently in renewable energy^4^, pharmaceutics^5^,^6^, agriculture^7^, and environmental remediation industries^8^. More recently, applications of mixed bacterial populations are being sought for commercial use, drawing on the ability of communities to perform a larger variety of more complicated tasks than individual species^9^. A major challenge in utilizing multiple bacterial species cooperatively in a complex chemical transformation reaction is to establish temporal population stability and to preserve the spatial distribution characteristics within the system^10^,^11^. In consortia or mixtures, interspecies competition may lead to an imbalance in population distribution, reducing efficacy and yield, and may even result in a complete termination of the process if one of the species is eradicated.

Aromatic hydrocarbons are major industrial feedstock chemicals and also environmental pollutants, therefore their biotransformation is important for both chemical synthesis and remediation applications. Aromatic hydrocarbons are oxidized to higher value products (*e.g*. phenols, benzoates, phthalates, quinones, hydroquinones, dyes), and biotransformation has a higher conversion efficiency than chemical transformation. However, the higher oxygenation demand of biotransformation makes it economically unfavorable for synthesis of these products. On the other hand, aromatic hydrocarbons with polycyclic rings (PAHs) are prominent carcinogens and EPA priority pollutants^12^, and therefore are top targets for water or soil remediation. Bioremediation of PAHs is shown to be feasible using *Pseudomonas* sp. NCIB 9816^13^, but the process requires continuous oxygenation via mechanical aeration. Aeration can account for more than 50% of the overall energy consumption in a typical bioremediation processes^14^, making bioremediation economically less feasible.

In this communication, we designed and produced a synthetic self-sustaining ecosystem where cyanobacteria, *Synechococcus elongatus* PCC 7942, provided the necessary oxygen for NCIB 9816 to biotransform aromatic hydrocarbons, eliminating external oxygenation requirement. PCC 7942 and NCIB 9816 were encapsulated in an optically transparent, highly porous, and mechanically sturdy silica gel for synergistic biotransformation of naphthalene to CO_2_ (Fig. 1a). Silica gel encapsulation of whole cells (eukaryotes and prokaryotes) has been studied in the literature, and is proven to be an effective tool for utilizing and studying cells in physical confinement, as summarized in review articles^15^,^16^. NCIB 9816 is known to aerobically transform over 100 aromatic hydrocarbons^17^, thus has a wide range of potential applications in chemical synthesis and remediation.

We engineered the system to maximize the combined activity of NCIB 9816 and PCC 7942 populations, while ensuring ease of integration into existing industrial processes. In almost all bioencapsulation systems, cells constitute the major proportion of the overall cost. Thus, encapsulated cell densities were optimized to maximize the biotransformation rate while minimizing the required amount of cells. The silica gel was further optimized for mechanical stability, cytocompatibility and optical transparency. Light attenuation (due to absorption and scattering effects of the encapsulating gel and the cells within) in the silica gel matrix was modeled, and experimentally verified by measuring the oxygen generation rates of the encapsulated PCC 7942 at various cell densities. This model was used to determine the geometry-dependent critical cell density parameter, which maximized total oxygen generation rate. Using this parameter, loading density of NCIB 9816 was optimized and the biotransformation performance of the system was experimentally verified. The developed ecosystem was able to sustain biotransformation reactions after the dissolved oxygen in the medium was depleted. Furthermore, it was shown that photosynthetic oxygenation via co-encapsulated PCC 7942 in very close proximity to NCIB 9816 was more efficient than external aeration due to micron scale diffusion length-scale between the co-encapsulated cells. Confocal microscopy was used to verify homogeneous distribution of the cells within close proximity.

## Results and Discussion

### Material design and characterization

**Figure 1:**
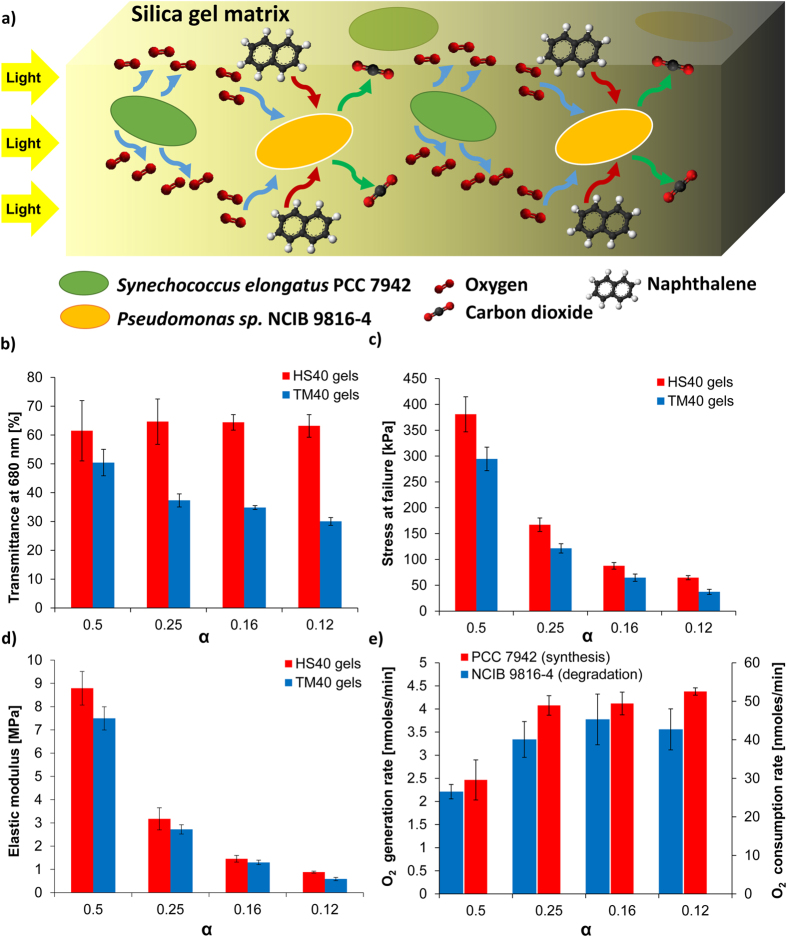
Silica gel matrix optimization based on its optical and mechanical properties, and the post-encapsulation activity of PCC 7942 and NCIB 9816. Four different α values (0.5: Highest Si alkoxide, to 0.12: Lowest Si alkoxide in gel formulation) and two different nanoparticle sizes (HS40: 12 nm, TM40: 22 nm) were tested. (**a**) Schematic of the biotransformation system illustrating the silica gel encapsulated bacteria, and the transport of substrates between cells (**b**) Optical transmittance of the gels at 680 nm and 1 cm pathlength, (**c**) Stress at failure, (**d**) Elastic modulus, (**e**) Oxygen generation rate of encapsulated PCC 7942 (in PBS) and oxygen consumption rate of encapsulated NCIB 9816 during biotransformation of naphthalene in saturated naphthalene solution *(All error bars indicate standard deviation with n* ≥ *3*).

Silica gels were synthesized using two precursors: Tetraethyl orthosilicate (Si alkoxide) and aqueous silica nanoparticles (SNPs). The volumetric ratio of the two silica precursors (α = Si alkoxide/(Si alkoxide + SNP)), and the diameter of the SNPs were varied to optimize the mechanical and optical properties of the silica gels. Optical properties of the silica gels varied considerably with the size of SNPs used. Gels made with HS40 (12 nm in diameter) and TM40 (22 nm) SNPs were visibly transparent, while NS85-40 (55 nm) and NS125-40 (85 nm) gels were opaque, independent of α. Since optical transparency is crucial for efficient oxygen generation, NS85-40 and NS125-40 gels were excluded from the study. Transparent gels synthesized with HS40 and TM40 SNPs were further characterized using UV-Vis spectrophotometry (Fig. 1b). In the entire photosynthetically active spectral range (PAR, 400–700 nm), transmittance (ratio of transmitted light to received light) in the gels synthesized with smaller (HS40) SNPs was higher than that of gels synthesized with larger (TM40) SNPs. The wavelength of 680 nm was selected for characterization studies as it is the maximum absorption wavelength for Photosystem II^18^, where oxygen evolving complex is located in cyanobacteria. It was further observed that transmittance of TM40 gels decreased gradually as α decreased, while that of HS40 gels were unaffected (Fig. 1b). This was attributed to formation of silica aggregates by the Si alkoxide during gelation^19^. These aggregates were smaller than the TM40 SNPs but comparable in size to the HS40 SNPs, thus they did not affect the optical properties of the HS40 gels.

The gel with the best mechanical properties (HS40/α = 0.5) had an elastic modulus of 8.79 ± 0.72 MPa and a stress at failure of 380 ± 34 kPa (Fig. 1c,d). HS40 gels had higher mechanical properties as compared to the TM40 gels, for all α values tested, which is due to the higher crosslinking density of the smaller nanoparticles. HS40 gels were also superior to TM40 gels in terms of optical transparency, thus TM40 gels were not further investigated. Mechanical properties (elastic modulus and stress at failure) of the gels declined as α decreased. This is in accordance with previously reported results that increasing Si alkoxide content of the gel improves its mechanical properties, albeit reducing the viability of the encapsulated biocatalytic bacteria^19^. In that system the encapsulated bacteria were recombinant *Escherichia coli* overexpressing a catalytic enzyme, and cell viability was not essential. However, in this system NCIB 9816 and PCC 7942 must both be viable to carry out biotransformation and photosynthesis, respectively. Thus, we proceeded with measuring their post-encapsulation biological activity with respect to α.

Activity of encapsulated PCC 7942 was evaluated by measuring its oxygen generation rate. The rate increased significantly when α decreased from 0.5 to 0.25, and plateaued around 4.4 ± 0.1 nmoles/min (Fig. 1e). Conversely, activity of the silica gel encapsulated NCIB 9816 was evaluated by measuring oxygen depletion in the supernatant due to biotransformation of a saturated naphthalene solution. The naphthalene-dependent oxygen consumption rate of encapsulated NCIB 9816 increased significantly from α = 0.5 to 0.25, and a maximum of 45.3 ± 6.6 nmoles/min was achieved at α = 0.16 (Fig. 1e). These results verified that a compromise was necessary between the cytocompatibility and the mechanical properties of the gel. HS40/α = 0.25 gels had very small loss in oxygen generation (with PCC 7942) and biotransformation (with NCIB 9816) activity, and maintained better mechanical properties as compared to the other cytocompatible (α = 0.16 and 0.12) gels. While the mechanical properties of all the tested silica gel formulations were sufficiently high for our experiments, we opted to use HS40/α = 0.25 gel for modeling and optimization purposes to facilitate its potential use in a scaled-up biotransformation application.

### Modeling of oxygen generation and consumption

A mathematical model was constructed to analyze oxygen generation by encapsulated PCC 7942 as a function of cell density (*ρ*) and distribution of light intensity (*I*) in the silica gel. Light intensity varies through the gel as both the silica gel and the encapsulated bacteria within contribute to light attenuation (*At*) via absorption and scattering. Spatial attenuation in one dimensional solutions in photobioreactors (where bacteria are freely suspended in media) has been extensively studied^20^, and is typically modeled by the Beer-Lambert law:


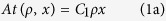


where attenuation coefficient *C*_*1*_ represents the contribution of cells to light attenuation, *ρ* is the cell density (of the photosynthetic bacteria), and *x* is the pathlength of light in the cell suspension. This model can be modified for silica gel encapsulated PCC 7942 as follows:


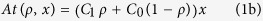


where attenuation coefficient *C*_*0*_represents the contribution of the gel to light attenuation, and *x* is the distance from the gel surface.

**Figure 2:**
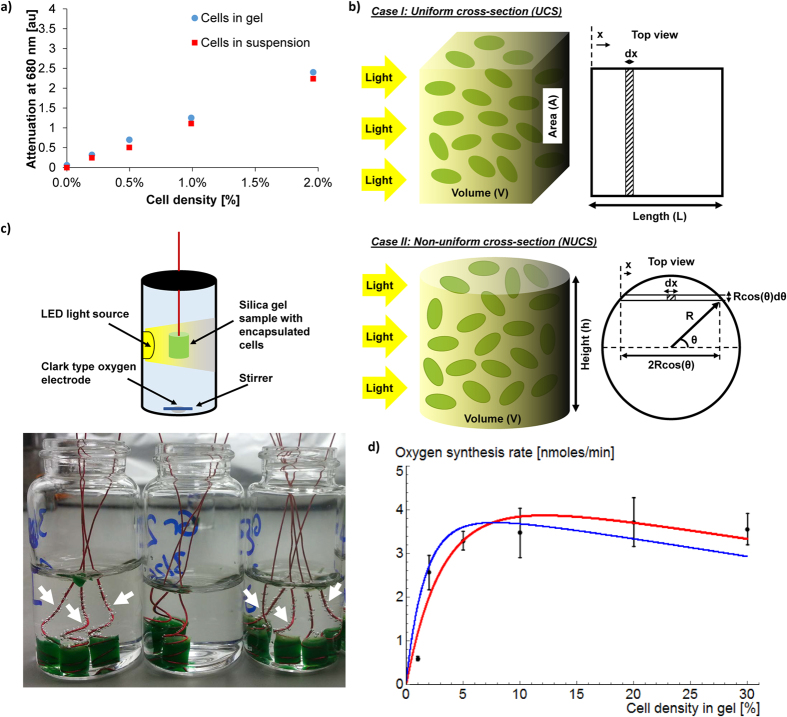
Modeling the oxygen generation rate of the silica gel encapsulated PCC 7942. Light attenuation in the matrix by the silica gel material and encapsulated cells was characterized using UV-Vis spectroscopy. Modeling results were experimentally verified by measuring oxygen generation rate of the encapsulated cells at varying cell density. (**a**) Light attenuation in cells suspended in PBS (red) and silica gel encapsulated cells (blue), (**b**) Schematic for the model in two different gel geometries with encapsulated cells, (**c**) Schematic: Experiment setup (Oxygraph) used for oxygen generation or consumption rate measurements with encapsulated cells, Image: Silica gel samples with encapsulated PCC 7942. Oxygen (synthesized via photosynthesis) bubbles are clearly visible on the supporting wires (indicated with white arrows), (**d**) Experimental measurements of oxygen generation rate of silica gel encapsulated PCC 7942 (black diamonds), and model results with (red curve) and without (blue curve) light back-scattering effects *(All error bars indicate standard deviation, n *> *3*).

Coefficients of Equation (1b) were determined experimentally by measuring light transmittance through HS40/α = 0.25 gels with encapsulated PCC 7942 at different cell densities, as well as with free PCC 7942 in suspension (Fig. 2a). It was observed that light attenuation through free and encapsulated cell volumes were very similar, indicating that the optical transparency of the designed gel (*C*_*0*_ = 0.089 1/cm) was sufficiently high and the major contribution to light attenuation in the gel was due to cells (*C*_*1*_ = 116.42 mL/(mL cells-cm)). Thus, the cell density and light penetration are inversely correlated and contribution of each bacterium to overall oxygen generation decreases along the pathlength (due to increased attenuation), potentially reaching zero at a critical distance from the surface. Using Equation (1b), transmittance (*T*) at a distance *x* from the gel surface for encapsulated PCC 7942 was determined as:


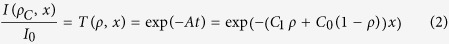


Note that *I*_*0*_ is the light intensity at the gel surface, which depends only on the light source characteristics. Since the light source in the experimental setup was fixed, light transmittance profile (*T = I/I*_*0*_) was used instead of the absolute light intensity (*I*) in the calculations, for simplicity.

Based on Equation (2), two volume elements (Fig. 2b) for cells encapsulated in a silica gel of uniform cross-section (UCS) and non-uniform (cylindrical) cross-section (NUCS) were defined. The UCS volume element was used for the derivation of an analytical solution of the critical cell density parameter and optimization of the cell densities for the synergistic biotransformation study. The NUCS volume element was used for the characterization of oxygen generation by the encapsulated cells in the Oxygraph chamber, where cylindrical samples were used. For UCS, a differential volume can be defined as:


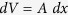


where *A* is the uniform cross-sectional area (the differential volume definition and the derivation of the following equations for NUCS is provided in the supplemental information). Oxygen generation rate of cyanobacteria is known to be linearly correlated with light intensity up to a saturation limit where photo-inhibition effects start damaging the oxygen evolution complex^20^. The system was designed to operate below the saturation limit, so the net oxygen generation rate (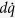) in the differential volume was defined as:


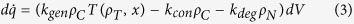


All the parameters used in Equation (3) are summarized in Table 1. The net oxygen generation rate in the volume was then obtained by integrating Equation (3) over the whole volume, and incorporating the consumption by the NCIB 9816, as shown in Equation (4):


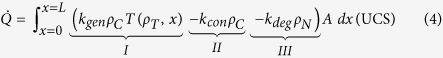


In Equation (4), (I), (*II*) and (*III*) represent the oxygen generation by PCC 7942, oxygen consumption by PCC 7942, and oxygen consumption during aerobic transformation by the NCIB 9816, respectively. Note that in the case when only PCC 7942 was encapsulated, Equation (4) simplified to:


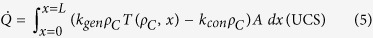


Critical cell density was evaluated by differentiating Equation (5) with respect to *ρ*_*C*_:


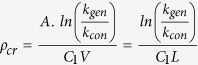


This result showed that critical cell density, *ρ*_*cr*_, was inversely proportional to the attenuation coefficient of the cells (*C*_*1*_) and path length of the light (*L*), as expected. *ρ*_*cr*_ thus yielded an upper limit for the net oxygen generation rate of the system. Critical cell density for NUCS geometry was numerically solved.

The results obtained from the mathematical model were verified experimentally using an oxygen electrode as shown in Fig. 2c. Cell density (*ρ*_*C*_) was varied from 1% to 30% [v/v] in the silica gel with PCC 7942 to determine how *ρ*_*C*_ affected oxygen generation rate (the contribution of the thin copper wire to light attenuation in the samples is assumed to be negligibly small). The net oxygen generation rate of the whole gel increased with increasing *ρ*_*C*_ up to a certain point, then slightly decreased (Fig. 2d). The maximum oxygen generation rate of 3.7 ± 0.7 nmoles/min was achieved at *ρ*_*C*_ = 20% [v/v]. Base oxygen consumption rate constant of PCC 7942 (*k*_*con*_) was measured in the absence of light (in the absence of oxygen generation) as 22.1 ± 1.7 nmoles/(min.mL-cells). Incorporating *k*_*con*_ and other known experimental parameters for encapsulated cells into Equation S3, *k*_*gen*_ was determined by least-squares regression with the experimental data. This yielded a *k*_*gen*_ value of 1228.6 nmoles/(min.mL-cells) for a fit as shown in Fig. 2d (blue line), and a *ρ*_*cr*_ value of 7.82% [v/v]. It was shown in the literature that cells receive some back-scattered light from other cells, which can also contribute to photosynthesis^21^. Thus, back-scattering contributions were incorporated into Equation (1b) with a coefficient *C*_*S*_, as follows:


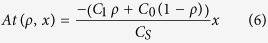


When back-scattering effects were accounted for *k*_*gen*_was 721.74 nmoles/(min.mL-cells), *ρ*_*cr*_ was 12.1% [v/v], and *C*_*s*_ was 1.88 with a fit as shown in Fig. 2d (red line). It is evident that the model, which incorporated back-scattering fit the experimental data and predicted the value of *ρ*_*cr*_ significantly better. The modified model was then used to optimize the relative densities of the co-encapsulated cells for naphthalene biotransformation.

### Optimization of the co-encapsulation matrix and synergistic biotransformation study

**Figure 3:**
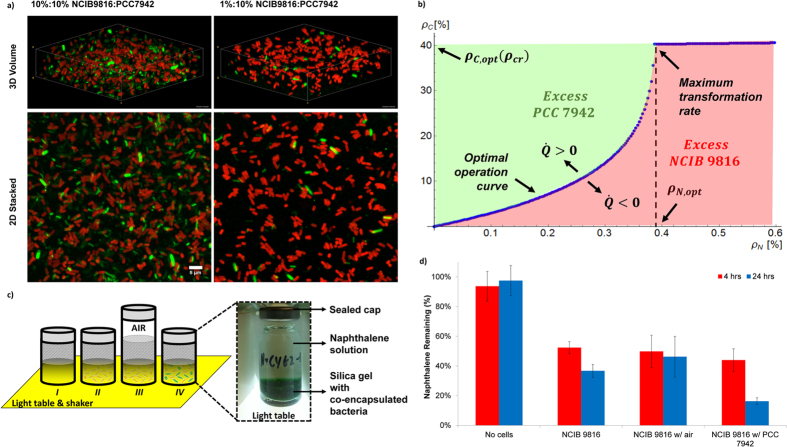
Synergistic biotransformation by silica gel co-encapsulated NCIB 9816 and PCC 7942. **(a**) Confocal images of silica gel co-encapsulated PCC 7942 (red) and NCIB 9816 (green) cells. Both species are homogeneously distributed in the silica gel matrix and positioned in micron-scale proximity. (**b**) Cell densities of PCC 7942 (*ρ*_*C*_) and NCIB 9816 (*ρ*_*N*_) optimized for the experimental setup of the biotransformation experiment. 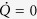 curve indicates the optimal operation conditions where the system has neither an oxygen deficit or surplus. The maximum biotransformation rate is achieved at *ρ*_*C*_ = *ρ*_*cr*_ and corresponding *ρ*_*N*_ on the 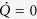 curve **c)** Schematic of the experiment setup used for biotransformation of naphthalene. Four cases were tested: I) No cells (Negative control), II) NCIB 9816 (Oxygen is limited to the dissolved oxygen in solution), III) NCIB 9816 with headspace (Supplemental oxygen is provided via the air in the headspace), IV) NCIB 9816 with PCC 7942 (Oxygen is provided by the co-encapsulated PCC 7942) **(d**) Results of the naphthalene biotransformation experiment. NCIB 9816 with co-encapsulated PCC 7942 achieved the highest biotransformation ratio (*All error bars indicate standard deviation, n *= *3*).

In mechanical aeration, oxygen partitions into water at the air interface, diffuses through the bulk of the liquid and the encapsulation matrix and reaches the biotransforming bacteria. On the other hand, encapsulated PCC 7942 generated oxygen in close vicinity of the biotransforming bacteria, NCIB 9816, minimizing transport barrier, enhancing the activity of the system developed here. Homogeneous distribution and micron-scale proximity of co-encapsulated cells in the gel matrix are illustrated by confocal microscopy images taken at two different ratios of PCC 7942 and NCIB 9816 (10%:10% [v/v] and 10%:1% [v/v]) (Fig. 3a).

The goal of the optimization study was to maximize the biotransformation rate of naphthalene to CO_2_ while minimizing the required amount of cells. We proposed that under optimal operating conditions, the system is to have neither an oxygen surplus (*i.e*. excess PCC 7942) nor an oxygen deficit (*i.e*. excess NCIB 9816). This design requirement was satisfied by solving Equation (4) for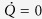, which yielded a non-linear *ρ*_*C*_ vs. *ρ*_*N*_ curve (Fig. 3b). It can be seen that *ρ*_*C*_ increased with increasing *ρ*_*N*_, up to *ρ*_*N*_ = 0.4% [v/v] where a *ρ*_*cr*_ value of 40% [v/v] was reached. If *ρ*_*N*_ is increased beyond this critical point, additional PCC 7942 cannot provide more oxygen for the biotransformation reactions, yielding an oxygen deficit (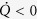).

Model analysis (in the previous section) showed that maximum oxygen generation rate was achieved at a critical PCC 7942 cell density: *ρ*_*C*_* = ρ*_*cr*_. It is clear that in order to maximize the biotransformation rate of the system, the density of PCC 7942 should be set to *ρ*_*cr*_, and based on the optimization study the density of NCIB 9816 should be at *ρ*_*N*,_ which yields 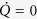. It should be noted that the solution which maximizes the biotransformation rate is not the most cost-effective point to operate the system, due to the non-linearity of the 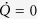 curve. Decreasing *ρ*_*C*_ and *ρ*_*N*_ (*i.e*. moving left on the 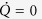
curve) would increase the cost-effectiveness of the system, but also reduce the biotransformation rate, which may not satisfy the performance requirements.

Based on the experimental parameters selected, the optimal volumetric ratio of NCIB 9816 to PCC 7942 were approximately 1/100 = 0.01. The efficiency of oxygenation can be calculated by considering a hypothetical case with no light attenuation in the gel (either by the gel or cells), where all the PCC 7942 generate oxygen at the maximum rate (*k*_*gen*_). In such a case, the NCIB 9816 to PCC 7942 cell ratio would be equal to *k*_*gen*_*/k*_*deg*_ = 0.04. Since 0.04 is the highest that this ratio can be, an efficiency factor can be calculated as: *η* = 0.01/0.04 = 25%. This result means that if the cells were co-encapsulated in an extremely thin gel with a single cell layer, one fourth of the encapsulated PCC 7942 would be sufficient to supply the same amount of oxygen. In our experiment setup, the strong hydrophilic interactions between the gel and the glass surface made it difficult to obtain a very thin gel with uniform thickness. Thus, experimental parameters were kept constant despite the low oxygenation efficiency.

Naphthalene biotransformation experiments with silica gel co-encapsulated cells were conducted as shown in Fig. 3c. Four cases were tested: (I) No cells: Silica gel without cells, (II) NCIB 9816: Silica gel encapsulated NCIB 9816, (III) NCIB 9816 with air: Silica gel encapsulated NCIB 9816 with additional headspace which provides additional oxygen for biotransformation reactions and (IV) NCIB 9816 with PCC 7942: Silica gel co-encapsulated NCIB 9816 and PCC 7942. Based on oxygen consumption experiments of a scaled-down system, the point where the dissolved oxygen concentration in the solution was expected to be depleted was approximately 4 hours. Thus, the time points were selected as 4 and 24 hours for the naphthalene biotransformation experiment. In Case I, naphthalene concentration decreased only slightly over 24 hours (Fig. 3d), verifying that the disappearance of naphthalene due to effects other than biotransformation was minimal. For cases II, III and IV, naphthalene concentrations were comparable after 4 hours at 52.3 ± 4.1%, 49.8 ± 10.9% and 43.9 ± 7.6% respectively. These values indicated that when a sufficient amount of dissolved oxygen was present in the solution, oxygen generation by encapsulated cyanobacteria did not make a difference. At 24 hours, the naphthalene concentrations in cases II and III were still comparable, whereas the naphthalene concentration for case IV was measured as 16.3 ± 2.3%, which was significantly lower than cases II and III. This indicated that dissolved oxygen was depleted in solution sometime between 4 and 24 hours, and after the depletion of dissolved oxygen, PCC 7942 provided oxygen to further drive the biotransformation reactions.

Experimental results also verified that oxygenation via co-encapsulated PCC 7942 (Case IV) was more efficient than providing oxygen externally (Case III). Oxygenation of a bioreactor using photosynthetic microorganisms was previously tested in spatially unstructured environments^22^,^23^,^24^, but was shown to limit the biotransformation rate. In our system, we observed the opposite result due to the optimized and stabilized cell densities, as well as close spatial distribution of the cells. It should be noted that an aeration system which provides air directly into the solution could perform better than Case III, since it eliminates the partitioning limitation of oxygen from air to solution. However, this condition was not tested since naphthalene is a very volatile chemical and ensuring that it would stay in the solution in an open system was not feasible. In our experiment, approximately 60 to 70% of the naphthalene was transformed with the amount of dissolved oxygen in water (Case II, Fig. 3d). Dissolved oxygen in water at room temperature (260 μM) can be utilized to transform approximately 15% of a saturated (246 μM) naphthalene solution into CO_2_ (based on 7.5 moles of O_2_ per mole of naphthalene as previously reporte^25^). This suggests that some partial transformation of naphthalene to intermediate species occurred without complete transformation.

Densities of PCC 7942 and NCIB 9816 were optimized based on their activity immediately after encapsulation, thus the biotransformation activity of the system was measured in a short time period (24 hours). It is known that the activity of encapsulated bacteria can vary over time, even when cell growth in the encapsulation matrix is restricted^26^. In addition, in our particular case a mutualistic relationship is expected to form between PCC 7942 and NCIB 9816, since CO_2_ is essential for carbon fixation by PCC 7942. Thus, oxygen generation by PCC 7942 will be regulated by the biotransformation rate of NCIB 9816, and vice versa. It is expected that due to the temporal changes in activity and formation of a mutualistic relationship, the optimal cell densities can change during long-term use of the system. However, cell densities can still be optimized using the tools presented in this study, depending on the specific biotransformation (e.g. synthesis yield, degradation rate, etc.) goals of the application.

In summary, in this study we developed a synthetic ecosystem via 3D co-encapsulation of two bacterial populations in a silica gel matrix for synergistic biotransformation. This technology has various immediate applications in chemical synthesis and environmental remediation, but could also be used in biomedical or biosensing applications. The potential to use silica gel encapsulated bacteria as biosensors is reported in the literature^27^,^28^, and co-encapsulation of multiple species can amplify this potential. We expect that co-encapsulated species could either act as a supporting organism to the primary sensory species, or work successively to biotransform a sensory input (*e.g*. chemical) into a signal (*e.g*. light). This technology could also be utilized as a platform to study fundamental microbial behavior. It has been long known that bacteria can act as a community via quorum sensing, and more recently interspecies quorum sensing was reported^29^. A synthetic ecosystem could provide a unique method to study this communication by enabling precise adjustment of bacterial communities’ volume and proximity.

## Methods

### Materials

Reagent grade tetraethyl orthosilicate (TEOS, 98%) and Ludox HS-40/TM-40 colloidal silica nanoparticles (SNP) were purchased from Sigma-Aldrich (Sigma-Aldrich Corp. St. Louis, MO, USA.). NexSil 85-40/125-40 silica nanoparticles were purchased from Nyacol (Nyacol Nano Technologies Inc., Ashland, MA, USA). All chemicals were used without further purification. Ultrapure water (UPW) was used in all the experiments, which was prepared by filtering distilled water through a Milli-Q water purification system (Millipore, Billerica, MA, USA) to a final electrical resistance of >18.2 MΩ/cm.

### Bacterial strains and growth conditions

*Synechococcus elongatus* PCC 7942 was obtained from the Pasteur Culture Collection of Cyanobacteria, France. *S. elongatus* PCC 7942 was grown at 28 °C in BG-11 medium^30^ and was contiguously bubbled with air. The cultures were incubated at an average light intensity of 50 μmol photons m^−2^ s^−1^. Cell growth was monitored by measuring OD730 on a Beckman DU 640 spectrophotometer (Beckman Coulter, Fullerton, CA). When the culture reached OD730 of 0.8, it was harvested by centrifugation at 8,000 rpm for 20 min at 22 °C. Cultures of *Pseudomonas sp*. NCIB 9816-4 were grown on Luria Broth (LB) at 30 °C for about 8 h and used to inoculate minimal salts buffer (MSB) at OD600 of 0.01. MSB was prepared following previously discussed methods^31^, with the following substitutions (Hutner’s Metals): 318 mg of Na_2_EDTA · 2H_2_O, 24 mg of CoSO_4_ · 7H_2_O, 17.7 mg of Na_2_B_4_O_7_ · 10H_2_O. The MSB was supplemented with 1 g naphthalene per 300 mL media. Cultures were grown in 2 L shake flasks (at 230 rpm) for 18 h at 25 °C with vigorous aeration. Cultures that reached a final OD600 of 1.5–2.5 were filtered through glass wool to remove any naphthalene crystals remaining in the solution prior to harvest. Cells were harvested by centrifugation at 5,000 g for 10 min. All cells were re-suspended at approximately 0.5 g wet mass/mL in phosphate buffered saline (PBS) for encapsulation. The OD730 of the cell suspensions were 1.2 after a 100-fold dilution.

### Silica gel synthesis and encapsulation of bacteria

Tetraethyl orthosilicate (TEOS) was hydrolyzed by stirring 2 h at a 1:5.3:0.0013 molar ratio of TEOS:water:HCl. The pH of the SNPs was adjusted to 7.4 by adding 1 M hydrochloric acid. After pH adjustment, PBS was added to further stabilize the pH of the SNPs and improve overall cytocompatibility of the solution. Bacteria suspension was added to SNP/PBS solution, and hydrolyzed TEOS was added to the SNP/PBS/bacteria solution by pipetting a few times to obtain a homogeneous sample. The final product was placed in glass vials or 96-well plates (depending upon the experiment) for gelation. Final volume of the gels consisted of 40% of silica precursors (TEOS + SNP) and 60% of (PBS + Bacteria suspension). Gels were named based on the volumetric ratio (α) of TEOS to (TEOS + SNP) in the gel formulation. For instance, SNP/α = 0.25 gel with 10% [v/v] cells had 300 μL of SNP, 100 μL of hydrolyzed Si alkoxide, 100 μL of cell suspension and 500 μL of PBS per mL of gel.

### Mechanical property measurement

Gels were synthesized in cylindrical molds (diameter = 12.5 mm, height = 12.5 mm). After gelation, samples were removed from the molds and placed briefly into PBS to keep them hydrated until testing. The samples were tested by axial compression until failure at a loading rate of 1 mm/min using an MTS QT10 mechanical testing machine (MTS Systems, Eden Prairie, MN). The stress at failure was reported as is, and elastic modulus was calculated from the linear-elastic region of the stress–strain curve using Matlab (Mathworks, Inc., Natick, MA).

### Oxygen generation/consumption and biotransformation rate measurement

Oxygen generation and consumption rates of the silica gel encapsulated bacteria were measured by using an Oxygraph Oxytherm System (Hansatech Instruments Ltd., United Kingdom). Silica gels were made into cylindrical 200 μL test pieces by mixing the reagents and bacteria suspension in a 96-well plate. After gelation, a thin copper wire was used to extract the test pieces (radius = 0.32 cm and height = 0.62 cm) and suspend them in the reaction chamber during measurement. For oxygen production measurements with cyanobacteria containing gels, 3 mL of PBS was added to the chamber and the LED was turned on to provide light to the cyanobacteria. The light intensity in the chamber was 89 μmol photons m^−2^ s^−1^ (estimated using pixel intensity and calibration from a light meter). For oxygen consumption measurements with NCIB 9816 containing gels, 3 mL solution of PBS saturated with naphthalene was added to the chamber.

For biotransformation rate measurements, NCIB 9816 and PCC 7942 were mixed gently via vortexing prior to encapsulation. Silica gels with co-encapsulated cells were synthesized (500 μL volume, height = 0.15 cm) in septa sealed, glass vials. After gelation, 9 mL of saturated naphthalene + BG-11 solution was added on top of the gel, ensuring that the vial was completely filled before sealing (except for the samples with additional 4 mL of headspace). Then, the samples were placed on a shaker setup and incubated at an average light intensity of 98 μmol photons m^−2^ s^−1^. Naphthalene concentrations in the solution were measured intermittently using gas chromatography–mass spectrometry, as described in a previous report^13^.

### Confocal imaging of co-encapsulated cells

NCIB 9816 were incubated with BODIPY R6G fluorescent dye (ThermoFisher Scientific, MA, USA) before encapsulation. PCC 7942 were encapsulated without addition of a fluorescent dye because of their auto-fluorescence. Samples were prepared by pipetting 20 μL gel volume on a glass slide and were sealed using an adhesive spacer and a cover slip. Confocal images were taken using a Nikon A1Rsi inverted confocal microscope (Nikon Instruments Inc., Melville, NY), using a 488 nm laser.

## Additional Information

**How to cite this article**: Mutlu, B. R. *et al*. Silica ecosystem for synergistic biotransformation. *Sci. Rep*. **6**, 27404; doi: 10.1038/srep27404 (2016).

## Supplementary Material

Supplementary Information

## Figures and Tables

**Table 1:** Oxygen generation rate model parameters.

Symbol	Definition	Value	Units	Notes
*k*_*gen*_	O_2_ generation rate constant per unit volume of PCC 7942 per s at 100% light intensity	721.73	nmoles/(min-mL cells)	Evaluated by fitting experimental data into the developed model
*k*_*con*_	O_2_ consumption rate constant per unit volume of PCC 7942 per s	22.1 ± 1.7	nmoles/(min-mL cells)	Experimentally measured via Oxygraph
*k*_*deg*_	O_2_ consumption rate constant during naphthalene transformation per unit volume of NCIB 9816 per s	~18,000	nmoles/(min-mL cells)	Adapted from Sakkos *et al*.^25^
*ρ*_*T*_*, ρ*_*C*_*, ρ*_*N*_	Cell density (T: Total, C: PCC 7942, N: NCIB 9816)	Varies	mL cells/(mL (gel) volume) % [v/v]*	ρ_C_, ρ_N_: Known ρ_T_ = ρ_C_ + ρ_N_
*C*_*0*_*, C*_*1*_*, C*_*s*_	Constants for contribution of cells (C_0_), silica gel (C_1_), and back-scattering effects (C_S_) to light attenuation	C_0_ = 0.089, C_1_ = 116.42, C_s_ = 1.88	[1/cm], [mL/(mL cells-cm], [dimensionless]	C_0_, C_1_: Experimentally measured via UV-Vis spectroscopy C_S_: Evaluated using the model

^*^Cell volume (mL cells) was equal to the volume of PBS suspended cells. See methods for details of wet cell mass in a given cell volume.
