# Supplementary material for: Silica ecosystem for synergistic biotransformation
Source: Sci Rep. 2016 Jun 6;6:27404. doi: 10.1038/srep27404 (PMC4893658; doi:10.1038/srep27404)
Supplement: Supplementary Information [file srep27404-s1.pdf]

Silica ecosystem for synergistic biotransformation

Baris R. Mutlu, Jonathan K. Sakkos, Sujin Yeom, Lawrence P. Wackett, Alptekin Aksan

Supplementary Information

Derivation of oxygen generation and consumption model for NUCS volume

For a cylindrical volume with a non-uniform cross-section (NUCS) as shown in Figure 2b, a differential volume can be defined as:

$$dV = R\,cos(\theta)\;h\;dx\;d\theta \text{ (NUCS) } \textbf{(S1)}$$

where R is the radius and h is the height of the NUCS differential volume. Then Equation (4) and Equation (5) is replaced by:

$$\dot{Q} = 2\int_{\theta=0}^{\theta=\pi/2}\int_{x=0}^{x=2Rcos(\theta)} (\underbrace{k_{gen}\rho_C T(\rho_T, x)}_{I}\ \underbrace{-k_{con}\rho_C}_{II}\ \underbrace{-k_{deg}\rho_N}_{III})R\;cos(\theta)\;h\;dx\;d\theta \text{ (NUCS) } (\mathbf{S2})$$

$$\dot{Q} = 2\int_{\theta=0}^{\theta=\pi/2}\int_{x=0}^{x=2Rcos(\theta)} (k_{gen}\rho_C T(\rho_C, x) - k_{con}\rho_C)R\;cos(\theta)\;h\;dx\;d\theta \text{ (NUCS) } (\mathbf{S3})$$
